# Tailoring robot-assisted arm training to individuals with stroke: bridging neuroscience principles and clinical practice

**DOI:** 10.3389/fneur.2025.1506889

**Published:** 2025-01-29

**Authors:** Giovanni Morone, Marco Tramontano, Stefano Paolucci, Antonio Cerasa, Irene Ciancarelli, Alex Martino Cinnera, Marco Iosa, Rocco Salvatore Calabrò

**Affiliations:** ^1^Department of Life, Health and Environmental Sciences, University of L’Aquila, L’Aquila, Italy; ^2^San Raffaele Sulmona, Sulmona, Italy; ^3^Department of Biomedical and Neuromotor Science, University of Bologna, Bologna, Italy; ^4^Unit of Occupational Medicine, IRCCS Azienda Ospedaliero-Universitaria di Bologna, Bologna, Italy; ^5^IRCCS Santa Lucia Foundation, Rome, Italy; ^6^Institute of BioImaging and Complex Biological Systems, National Research Council (IBSBC-CNR), Catanzaro, Italy; ^7^Sant'Anna Institute, Crotone, Italy; ^8^Department of Psychology, Sapienza University, Rome, Italy; ^9^IRCCS Bonino Pulejo Neurology Center, Messina, Italy

**Keywords:** robotic devices, exoskeleton devices, stroke, rehabilitation, upper extremity

## Abstract

Robot-assisted arm training (RAAT) has demonstrated promising potential in improving rehabilitation outcomes for individuals with neurological conditions, particularly stroke. Despite 20 years of their use in clinical and research settings, there are still significant needs to be made concerning clinical indications. In the present perspective manuscript, we provide some hypotheses of the suitability of different RAAT according to the features of the available devices and clinical characteristics, showing their limitations and strengths. Several factors were considered in the optimization of RAAT intervention, including the technological characteristics of the devices (e.g., support and constriction), the residual upper limb motor function, and the clinical phase of stroke. Finally, we outline key areas for improvement to advance the field in the near future and provide neuroscientific bases for hypotheses of tailored RAAT training to improve the outcome of robotic rehabilitation.

## Introduction

Robot Assisted Arm Training (RAAT) has long been envisaged as a strategy to enhance arm motor recovery after a stroke (1). Significant efforts have been made toward identifying the neural mechanisms underlying RAAT and their relationship with improved motor recovery (2–5). The rationale behind the application of robots in stroke rehabilitation is that RAAT involves areas of the brain that govern movement planification and execution by early, standardized, repeatable and intense mobilization of the patient’s arm (6, 7). Such reiterated engagement of motor areas is intended to influence brain plasticity phenomena, improving functional outcomes (8). The role of an early mobilization after stroke is well recognized in literature to reduce acute phase complications and improve functional outcomes, especially to more severely affected people (9).

Interestingly, to be task-oriented the RAAT often embedded a system for serious-game technology on which a digital task runs allowing the patient to interact with it, for providing to him/her the execution of task oriented movements and giving visual and acoustic feedback of his/her performance. Despite robots and serious videogames (implemented using virtual reality or on a screen) are two clearly distinct technologies, the common combination in RAAT implies some motor and cognitive links that contribute to enhancing hand and arm sensory-motor areas (10), as well as cognitive functions (11) but makes more difficult to discriminate the specific effect attributed to the robotic system itself, leading to observe cognitive effects of upper limb robotic therapy (12, 13).

Nowadays, evidence for a clinical benefit of RAAT is consolidated as per Cochrane collaboration and in several stroke national guidelines (14, 15). Nonetheless a recent large, randomized, controlled trial in people with subacute stroke reported no significant clinical improvement of RAAT vs. enhanced upper limb therapy (16).

There are different devices for the upper limb that can be distinguished firstly in electromechanical devices vs. robots (being the second adaptable to the patient’s motor behavior during the task), on the basis of clinical characteristics (interactive/assistive), based on technical characteristics (Exoskeleton/End-effector, number of active and/or passive degree of Freedom, Bilateral/Uilateral, distal/proximal, feedback, Wearability, Environment [Real, Virtual], Assistance modality, Recorded parameters [Range of motion, Force, Kinematics], Control system [Force, Range of motion, Impedance, EMG] and Movement dimension [1D/2D/3D]) (17–19).

The main point to be improved regarding the RAAT, and in general to the translational sciences of the robots applied to rehabilitation, is to define the best practices related to the clinical characteristics of individuals that might benefit from a specific type of robot (i.e., exoskeleton/end effector, constrictive characteristics, degree of freedom, cognitive load, movement intention detection, and more).

Although physicians and customers have been looking for solutions to the robotic clinical protocol problem for decades, it is still challenging to find the best candidates for RAAT. Actually, assessing the literature by itself is insufficient to reach this conclusion because each RCT is biased by its design, which assesses overall efficacy rather than the beneficiary’s functional characteristics. According to the Precision Medicine Initiative, that promotes an emerging approach for disease treatment and prevention that considers individual variability (20), and to previous studies that changed the research question from “is robotic treatment effective?” into “who may benefit from robotic therapy?” (21–23), we hypothesized possible indications on the use of RAAT considering rehabilitation objectives, persons functional characteristics, and time from stroke acute event. This manuscript aims to provide neuroscientific bases for hypotheses of personalized robotic training.

Specifically, we will analyze some fundamental aspects that need to be considered from researchers and clinicians for a better integration of robotic training in the rehabilitation project of individuals with stroke. We will analyze the characteristics of the machine according to the patient’s motor possibilities, the cognitive-motor interactions during the execution of robotic functional tasks (24), the continuum of care through the different settings, the importance of contrasting the phenomenon of non-use learning, the functional cross talk between hand and upper limb recovery and finally the personalization of robotic therapy.

## Machine constriction vs. arm functionality: hypothesis for robot clinical use

The term **“machine constriction”** in the context of robotic devices for motor recovery in people with stroke likely refers to the physical and functional limitations that a machine, like a robotic rehabilitation device, can impose on a patient during therapy (17). These constrictions could involve:

1. **Physical restraints**: The robotic device might physically limit or guide the movement of a patient’s limb to ensure it follows a controlled range of motion. This can prevent unsafe or incorrect movements but may also feel restrictive if the machine over constrains the motion (17).
2. **Assistance level or weight support**: Some robotic devices may constrict the amount of assistance or resistance they provide during therapy. For instance, they may limit how much they aid or oppose the patient’s movements, depending on the patient’s ability to move on their own (17–19).
3. **Degree of freedom**: Robotic systems may have a limited number of degrees of freedom (DoF), meaning they allow motion only in certain directions or planes. This is a form of “constriction” that simplifies the movement patterns for rehabilitation but may not fully mimic the complexity of human movements (17–19).
4. **Best candidate**: Even if robotic devices have a wide margin of adaptability based on the functionality of the patient, they are built taking in mind a specific type of patient, which is the best candidate. That is, the ideal patient who, due to the specific phase of the event, cognitive and motor functional condition, can benefit from a specific robotic treatment (23).

In the context of stroke recovery:

- **Device constriction** can ensure **safe rehabilitation** by guiding a patient’s movement in a specific, therapeutically beneficial way. It can help train specific muscles and neural pathways damaged by the stroke.
- However, too much **constriction** can prevent the patient from using their muscles and motor control fully, limiting the effectiveness of the therapy and possibly hindering the recovery of motor independence.

A balance between assistance and freedom of movement is critical for optimizing motor recovery in people with stroke using robotic devices (23).

Using the *“from efficacy for all to all for efficacy”* approach (23, 25, 26), in this section we report the best practice for addressing patients to the best RAAT-related treatment. First, to maintain an intensive exercise with specific and timely tasks, in more compromised persons it is useful to use an exoskeleton that guarantees better motor control. When the patient recovers or if he/she already has some degree of muscle activity, end-effectors (that move the hand for moving the whole kinematic chain of the upper limb) can also be used (27, 28). In case of persons with mild to moderate impairment, sensor-based devices can be used (29). If the patient has a very mild disability, a conventional/ occupational approach with dedicated functional exercises in an ecological environment (without typical constriction provided by the robots) is often the best solution (2). This hypothesis of robot uses according to persons’ functionality is in line with the principles that in the face of a lower possibility of voluntary movement of the patient, a greater possibility of the robot to control and implement the movements of the upper limb and hand must correspond to a greater possibility of the robot (23, 26). Furthermore, this theory is consistent with recent research showing that persons who are more severely affected are the ones who stand to gain the most from robotic therapy (27, 30, 31).

It is of note that the relationship between intensity of training and recovery is not linear. Pila et al. recently analyzed responders vs. non-responder after RAAT noting that non responders do not improve arm functionality even in presence of an increase of RAAT intensity. Authors concluded that probably it is important to match training dose with robot parameters to allow a better functional recovery mediated by robot therapy (32). It is hypothesized that, despite the high intensity attained even in the more severely affected persons, the robot’s constriction is insufficient to achieve a clinically significant improvement in light of these results and the fact that arm sensory motor recovery depends on more than just training intensity.

It is also important to underline that a correct stratification of patients must inevitably consider the adaptability of the device to administer an exercise that leads to better performance during the exercise (i.e., increased participation, intra-session functional increase). Correct patient stratification and/or better robot identification will be the basis for creating evidence of greater robustness in the near future to avoid the mistakes made in the recent past. This section is well-articulated but could benefit from a more explicit discussion on patient stratification and device adaptability, particularly for future research directions.

**Figure 1:**
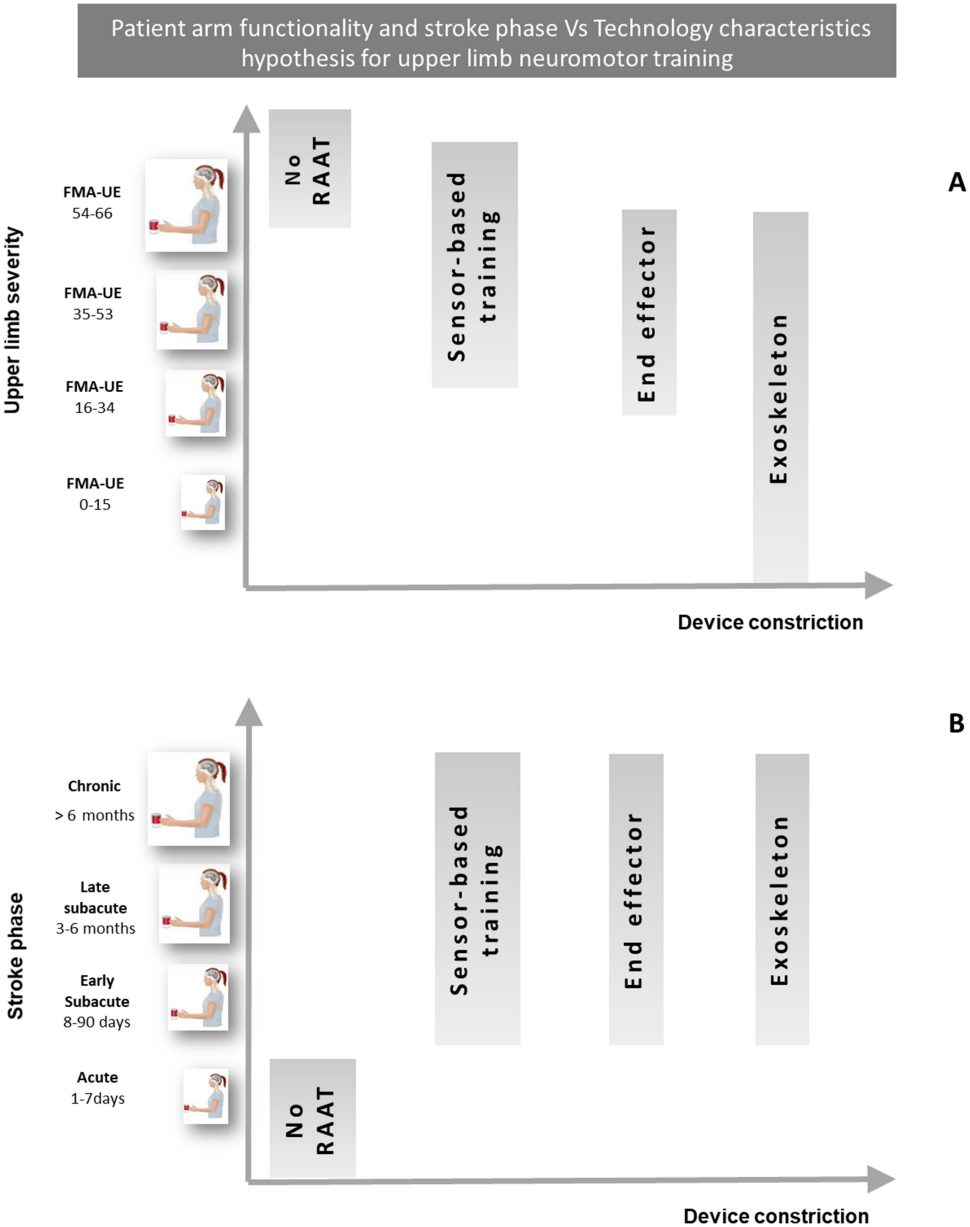
Hypothesis for the selection of RAAT to allow an adequate intensity of training, based on the technological characteristics of the devices and the functional status of the upper limb **(A)**, and on the clinical phase of stroke **(B)**. Adapted from Morone et al. (23). RAAT, robot assisted arm therapy; FMA-UE, Fugl Mayer assessment—upper extremities.

Figure 1 presents a graphical representation of a possible hypothesis of treating arm paresis with a robot, allowing adequate intensity even for more severe persons and according to stroke phase, thanks to the robot characteristics. The functional status of the upper limb was stratified using the Fugl-Meyer Assessment scale for the Upper Extremity (FMA-UE), where a higher score indicates better functionality. The classification of stroke phases followed the ESO guidelines for stroke rehabilitation.

To develop our treatment hypothesis, we stratified the arm severity presented in Figure 1A according the FMA-UE cut-offs proposed by Woytowicz et al. (33) clustering the UL functional level as follow:

- Severe (0–15): no hand, wrist, or multi-joint movements and limited to no movement from single joint extensor and flexor muscle synergies.
- Severe–Moderate (16–34): marked impairment with no movement out of synergy and limited movement from single joint extensor and flexor synergies, hand, wrist, or multi-joint movements.
- Moderate–Mild (35-53): moderate impairment with limited movements out of synergy and partial impairment of single joint extensor and flexor synergies, hand, wrist, and multi-joint movements.
- Mild (54-66): minimal impairment and able to perform movements out of synergy with full movement of the arm.

Figure 1B shows the classification of stroke clinical phases following the ESO guidelines for stroke rehabilitation (34). It should be considered that in the acute and early subacute phase, functional impairment is certainly greater and therefore in this phase the use of robotic devices could be important, as it is crucial for the execution of complex tasks, and not only because neuroplasticity processes are more active (14, 15).

## Machine feedback vs. cognitive reserve: implication for functional motor recovery

Over the past ten years, many paradigms for upper limb robotic rehabilitation have been completely or partially shifted from Bottom-up to Top-down training (3, 35, 36). This evolution can be attributed to the integration of visual interfaces, both immersive and non-immersive, with robotic systems. These interfaces enable the conversion of passive movements into a diverse array of task-oriented exercises that offer performance feedback, thereby engaging multiple cognitive functions during the learning process. These real-time and goal-directed feedback are well known key components to guide motor relearning (36). Furthermore, feedback can be manipulated in different ways, e.g., with error-based or positive-reinforcement mechanisms, by augmented or multiple feedback, real-time or after-performance feedback (37). It is also possible to insert during therapy a biofeedback/neurofeedback, a powerful means to modulate dependent on the activity of sensorimotor cortical networks (38). The cognitive trigger is often allowed thanks to the countless feedback provided by the 2D screen (non-immersive virtual reality) or even sometimes with serious game content that elicits executive functions (memory tasks, attention, working memory, task switching) and helps to make rehabilitation more salient and motivating, eliciting neuroplasticity (39).

Patients’ interest can be further increased by combining immersive VR with RAAT (40). This scenario suggests that there will be more natural 3D feedback to the 3D arm workspace, together with a genuine sense of presence in the virtual world and the corresponding neurophysiological reactions (as shown by physiological reactions in terms of heart rate, blood pressure etc.) (41). During a training performed in a VR scenario, the first perspective of the avatar induces an embodiment and hence a sense of body ownership that might impact the way in which persons perform an action (42). Immersive VR has the potential to influence motor brain networks thanks to the possibility of modifying the perception of reality (43).

One unique and fascinating way to manipulate attention in addition to visual and auditory stimuli is through dual tasking, which can be done during robotic therapy. Because the patient struggles to manage two activities at once, this approach creates more challenging working conditions for them, but it has been demonstrated to be highly helpful for functional recovery (44). However, Ranzani and colleagues have recently shown that an equivalent dosage of neurocognitive training in people with subacute stroke does not result in less motor skills improvement than robot-assisted therapy of hand function using a neurocognitive approach (i.e., combining motor training with somatosensory and cognitive tasks) (45).

Dual tasking training is of particular interest in persons with disability from neurological origins for its role in motor performances and for predict cognitive decline (46–49), however in people with stroke and with cognitive deficits it might lead to a global improvement of the cognitive function, which was supported by the improved neural efficiency of associated brain areas (50).

## Continuum of care, subacute and chronic individuals, from hospital to home setting

An early discharge from hospital wards is necessary for the reduction of costs per individual patient and for an optimization of resources in rehabilitation of neurological conditions (51). Then, there is a need to treat patients at home or on the territory, once discharged from the subacute neurorehabilitation hospital, in continuity with what was done in the hospital. Telerehabilitation programs, for example, gave the opportunity to continue at home the task oriented and intensive training that was undertake in hospital (52). To this aim, robots can help, but smaller and more economic versions are needed to continue reaching the goals stated during the in-hospital training (53). The importance of continuum of care in upper limb and hand recovery is even greater than for other functions such as walking and balance. Indeed, upper limb function and the related activities of daily living may need clinically more time to recover, and important improvement may be observed also in the chronic phase. This scenario is supported by recent evidence that even in the chronic phase, intensive and task-oriented robotic training can lead to an improvement in motor control, and in some cases even a slight improvement in functionality (54–57). Continuing treatment is not only important from an economic point of view because it saves economic resources and makes care more efficient, but it is important from an ethical point of view, since patients can further improve functionally and in their quality of life. For technologies and robotics to play an important role in the scenario of the continuum of care, it will be necessary to plan easy-to-use, less expensive and more usable devices with simpler human-robot interfaces.

## Arm related learning non-use and the importance of the early arm training and of the bimanual exercises

In the 1990s, Taub thoroughly explained the phenomenon of learning that has since allowed us to understand various mechanisms of maladaptive plasticity (58). Clinically, this maladaptive plasticity manifests as non-use, pain, and spasticity. This phenomenon is significantly more noticeable in the upper limbs because many daily activities can be performed with just one arm, supplying affected arm/hand reduced functionality (59, 60). Starting from these neuroscientific principles, robotic rehabilitation should be promoted as soon as possible and with adequate intensity, affected arm movement with goal-oriented functional exercises (61). Furthermore, the addition of other devices such as virtual reality, transcranial stimulations, should provide highly personalized rehabilitation exercises to counteract the phenomenon of maladaptive plasticity and promote the recovery of bimanual activities (62). In fact, bimanual activities recovery depends on the extent of corticospinal tract injury and initial sensory and cognitive impairments (63) and are directly related to the patient’s return to work and to the people community life. This should be promoted not only thanks to bimanual robots (Exoskeleton or end-effector) (5), but even implementing, for example, unilateral robots with appropriate Augmented Reality/Virtual Reality bimanual training. Bimanual cooperation, in fact, plays a vital role in functions of the upper extremity and daily activities and re-training bimanual force coordination in stroke survivors could facilitate a higher degree of participation in daily activities (64, 65). It is of note that the integration of both hands in a rehabilitation task led to cross-education and bilateral transfer of sensory motor learning phenomena between less affected hand (i.e., non-affected hand) to more affected hand that might increase functional recovery (66, 67).

## Arm recovery and hand recovery, proximo-distal approaches vs. disto-proximal approaches

The hand and the upper limb are two very distinct body structures with very distinct functionality (mainly grasping and reaching, respectively) (68). To improve distal part of the arm (hand and wrist) and decrease spasticity of the whole upper limb, the treatment based on distal-proximal approach is more effective than that of proximo-distal one (69, 70) and even better if the treatment is enriched with cognitive contents and bilateral training (71). Although this assumption is so true and easy to understand, it is often neglected to be applied in clinical practice (72). Indeed, when using either conventional rehabilitation or robots, especially during subacute phase, we train mainly the lower limb and the proximal upper limb, and little space is dedicated to the hand (73).

However, hand function is complex, and manipulation is a distinct important feature of human beings. This is why the cortical representation of the hand is proportionally larger than those related to other body parts, as described recently following Penfield’s findings (74).

According to conventional rehabilitation techniques, supporting distal functions is essential. However, outcomes for hand recovery are generally poor and often not detailed in clinical trials. Recent evidence suggests that starting rehabilitation distally, particularly with advanced technologies, could immediately stimulate the cognitive and sensory-motor networks of the hand, leading to better representation and potentially improved outcomes. Our hypothesis is corroborated by the study of Calabrò and coworkers. They demonstrated that the treatment of distal part of the arm with a robot, in people affected by chronic stroke, improves motor function by rebalancing interhemispheric connectivity (8).

During neuromotor rehabilitation, it will undoubtedly be essential to construct an increasing number of hand-specific robots and devices and use them either in parallel or in series. In any case, an individual and personalized rehabilitation project must be able to provide the possibility of training the distal or proximal part of the limb depending more on the characteristics of the patient. In fact, they should not be understood as antithetical but synergistic approaches.

## Arm robot and rehabilitation of neurological conditions: shaping the future of persons’ functional recovery

**Figure 2:**
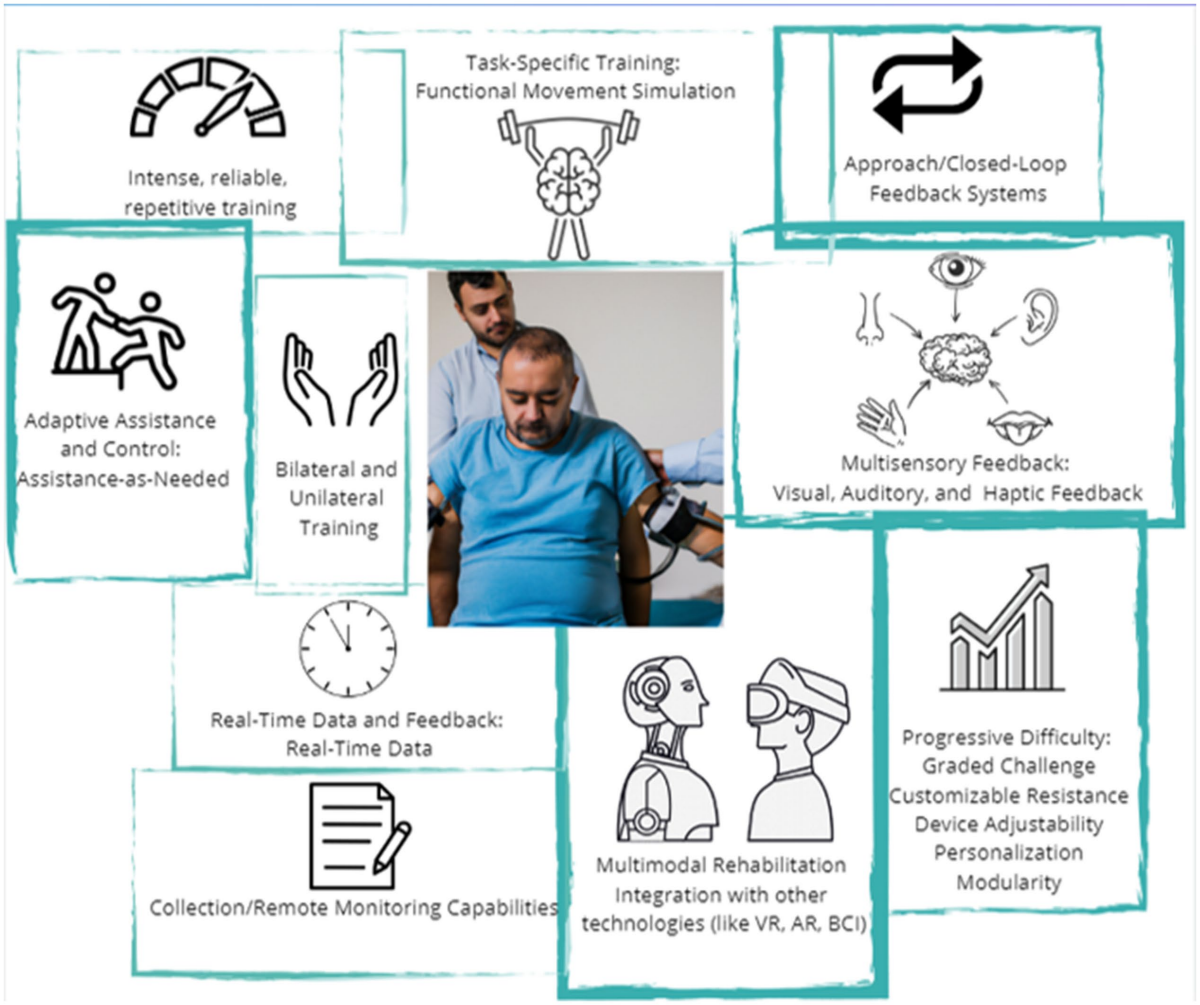
Key features of robotic devices for upper limb rehabilitation. Choosing the device based on its characteristics is fundamental to personalizing the treatment (this original figure was generated using AI tools).

Robots and technology aim to provide neuromotor rehabilitation for arm and hand recovery. They hold great potential for advancements and improvements in patient care (Figure 2) (14, 75, 76).

Therefore, comparing neuromotor therapy administered with robotic or electromechanical devices and conventional neuromotor therapy for the upper limb in people with stroke, current evidence affirms that: RAAT improves activities of daily living scores; improves arm function and arm muscle strength, and did not increase the risk of participant dropout (18).

Aspects to be investigated in the future concern the possibility of achieving the objectives earlier, which is important for the improvement of the cost-effectiveness parameters of healthcare and of the whole Health technology Assessment, HTA process. Clinicians as well as bioengineers should shape the future of this field before that trade decides products and markets. To reduce the machine constraint, which in some subgroups of persons represent a limitation, the use of wearable devices with semi-rigid or soft fabrics could broaden the range of patients who could benefit from RAAT. To date, the soft-robots and exosuit are not meeting the needs of their users (77), requiring future advancements in this way. Moreover, many robotic devices can be integrated into a closed-loop system, combining the robot with biosignal acquisition (e.g., EEG, EMG), allowing for reinforced learning by generating robotic movement when a functional activity is detected. Other aspects to carefully consider for this purpose include the integration of other technologies, particularly the virtual reality/augmented reality (VR/AR) and the artificial Intelligence (78) and the modularity of the new robot devices for the more patient centered adaptability.

The personalization of motor and cognitive rehabilitation therapy must not only be implemented on the patient’s impairment/functionality aspects but must also consider factors such as age (79), gender (80) and psychological and behavioral aspects (81) that are decisive for the acceptability and effectiveness of the therapy. Artificial intelligence can adapt exercises following the motor and behavioral information captured by the robot’s sensors during therapy (inertial sensor unit, force detection, facial expression recognition). The integration of robots with AR and VR technologies can enhance rehabilitation training improving the engagement, the motivation, and the interaction during training. Additionally, AR and VR can simulate real-life personalized scenarios (*ad hoc* per function to be improved, persons’ cognitive resources and persons’ behavioral issues), allowing patients to practice functional movements in a safe and supervised environment before discharge (82, 83). It is important to note that these technologies are always supervised by physiotherapists or occupational therapists (84) and incorporated into the individual rehabilitation program. RAAT will continue to evolve to provide more personalized and adaptive therapy. They will be able to assess a patient’s abilities and adjust the rehabilitation therapy, tailoring it to their specific needs in terms of motor and function assistance and cognitive load (85). The robot or therapeutic technological device should inform the therapist of a rapid decrease in performance to allow him/her to re-modulate the type of training or to limit the training, suspending it, in unsuitable therapeutic conditions. The robots should also provide information on the patient’s state of stress by means of sensors for recording autonomic parameters (86, 87). This is particularly suitable when robots are used with populations with reduced communication skills and/or with children. Continuous monitoring and assessment: Robots equipped with sensors and wearable devices will enable continuous monitoring of patients’ progress and functional abilities. This real-time data can be used to track changes, identify potential issues, and adjust therapy plans accordingly. Continuous monitoring can also facilitate early intervention and prevent complications (5). The integration of RAAT in the clinical practice will continue to complement the conventional training rather than replace them. They will assist therapists in delivering therapy, providing objective data, and automating repetitive tasks (85, 88). This integration will allow therapists to provide more supervised sessions of treatments and focus on more complex and individualized aspects of patient care. Robot adaptability for severity and for setting: for less affected patients to show that they are doing more salient therapy and for the most severe patients to demonstrate a better ability to intercept movement intentions (5). Economic sustainability: healthcare managers and insurance must give credit to robot-assisted neuromotor therapy by increasing financial reimbursements, as it improves outcomes or reduces hospitalization time. At the same time, rehabilitation processes and rehabilitation spaces need to be adapted, as robots require large spaces and trained personnel. Finally, there is the urgent need of a greater accessibility in terms of costs of robots and an increasing of the clinicians’ knowledge regarding robot integration in the rehabilitation process.

## Discussion

Robotic devices have been introduced into clinical practice, but they are not always used in accordance with the patient’s characteristics, resulting in reduced efficacy. Despite, the recent literature reported a significant general efficacy of using robots in rehabilitation of patients with stroke in adjunction, partial or total substitution of conventional therapy (14, 15, 18), some contrasting results is still observed (16) and it could be due to the selection of patients as candidates for robotic therapy. According to our mentioned hypotheses, we proposed that the choice of using a specific device should be based on the patient’s functional level, carefully taking into account both motor (e.g., exoskeletons for more severe patients and end-effectors for less severe patients) and cognitive (e.g., challenges in feedback) domains. In motor recovery, the different levels of weight support offered by various types of robots (complete, partial, counter-resistance) or the type of device (exoskeleton, end-effector, sensor-based) make it possible to personalize the treatment and optimize outcomes. The determining factors in the choice of devices are, from one side the objective of the robot/device assisted therapy and for another side the patient’s characteristics (i.e., residual motor-cognitive functionality, and phase of stroke, subacute or chronic). For devices, the characteristics to consider are the degree of constraint of the machine and the level of movement support, the kind and the scope of feedback interaction, the type of mobilization (distal-proximal or proximal-distal), and whether the robot is unilateral or bilateral. In our view, the matching of patient and device characteristics is the key to a personalized approach to RAAT. Looking to the future, the implementation of virtual reality, artificial intelligence, and wearable sensors, alongside the development of wearable soft-robots and closed-loop systems, could advance the use of RAAT with protocols tailored to individual needs. At the same time the merge of these different technologies will require specific researches to discriminate the combined effect of robotic devices with other systems (virtual reality, feedback, artificial intelligent control) vs. simple robotic devices. Finally, a large multicenter randomized trial with an appropriate stratification of people with stroke functionality for a given robot device to validate the proposed hypotheses is needed.

## Data Availability

The original contributions presented in the study are included in the article/supplementary material, further inquiries can be directed to the corresponding author.
